# A New Hydrogen Sensor Fault Diagnosis Method Based on Transfer Learning With LeNet-5

**DOI:** 10.3389/fnbot.2021.664135

**Published:** 2021-05-21

**Authors:** Yongyi Sun, Shuxia Liu, Tingting Zhao, Zhihui Zou, Bin Shen, Ying Yu, Shuang Zhang, Hongquan Zhang

**Affiliations:** ^1^Key Laboratory of Electronics Engineering, College of Heilongjiang Province, Heilongjiang University, Harbin, China; ^2^Department of Information, Liaoning Police Academy, Dalian, China; ^3^School of Automation, Harbin Engineering University, Harbin, China

**Keywords:** hydrogen sensor, fault diagnosis, transfer learning, LeNet-5, machine learning

## Abstract

The fault safety monitoring of hydrogen sensors is very important for their practical application. The precondition of traditional machine learning methods for sensor fault diagnosis is that enough fault data with the same distribution and feature space under the same working environment must exist. Widely used fault diagnosis methods are not suitable for real working environments because they are easily complicated by environmental conditions such as temperature, humidity, shock, and vibration. Under the influence of such complex conditions, the acquisition of sensor fault data is limited. In order to improve fault diagnosis accuracy under complex environmental conditions, a novel method of transfer learning (TL) with LeNet-5 is proposed in this paper. Firstly, LeNet-5 is applied to learn the features of the data-rich datasets of gas sensor faults in a normal environment and to adjust the parameters accordingly. The parameters of the LeNet-5 are transferred from the task in the normal environment to a task in a complex environment by using the TL method. Then, the migrated LeNet-5 is used for the fault diagnosis of gas sensors with a small amount of fault data in a complex environment. Finally, a prototype hydrogen sensor array is designed and implemented for experimental verification. The gas sensor fault diagnosis accuracy of the traditional LeNet-5 was 88.48 ± 1.04%, while the fault diagnosis accuracy of TL with LeNet-5 was 92.49 ± 1.28%. The experimental results show that the method adopted presents an excellent solution for the fault diagnosis of a hydrogen sensor using a small quantity of fault data obtained under complex environmental conditions.

## Introduction

With the gradual depletion of traditional energy sources and the development of clean fuel, clean fuel plays a prominent role throughout many fields (Tsujita et al., 2005; Brown et al., 2015). As hydrogen is a clean fuel, its application range is therefore rapidly expanding (Chalk and Miller, 2006). It is widely used in meteorological science, aerospace technology, the metallurgical industry, the electronics industry, national defense, the chemical industry, and so on, and its consumption is also increasing rapidly (Poirier and Sapundzhiev, 1997; Winter, 2005; Staffell et al., 2019). Hydrogen is a colorless, odorless, flammable, and explosive gas. It is necessary to monitor hydrogen concentrations using hydrogen sensors because it is considered a dangerous chemical (Song et al., 2019).

Semiconductor gas sensors have been widely used in hydrogen detection based on SnO_2_-sensitive materials (Fedorenko et al., 2017; Zhang Q. et al., 2018). However, they can be hindered by sensor aging, environmental temperature and humidity, sensitive material falling off the sensor, gas adsorption poisoning, and other factors that could affect the reliability of the sensors. Hydrogen sensors are prone to failure in its hydrogen safety detection function, which may lead to combustion and explosion. Therefore, hydrogen sensors' fault diagnosis is of great importance. Ingimundarson et al. proposed model-based detection of hydrogen leaks in 2008 (Ingimundarson et al., 2008). Ma et al. proposed hydrogen sensor for fault detection of power transformer in 2012 (Ma et al., 2012). Song et al. proposed a fault diagnosis and reconfiguration strategy for hydrogen sensor array in 2019 (Song et al., 2019). Sun et al. proposed a new convolutional neural network method for hydrogen sensor fault diagnosis in 2020 (Sun et al., 2020).

Recently, traditional machine learning (ML) methods have been widely used for fault diagnoses, such as the extreme learning machine (ELM) (Song et al., 2019), empirical mode decomposition (Chen Y. S. et al., 2016), support vector machines (SVM) (Hu et al., 2005), KNN (Yang et al., 2016b), non-negative matrix factorization (Yang et al., 2016a), gray forecasting (Chen Y. et al., 2016), learning vector quantization (LVQ) (Bassiuny et al., 2007), random forest (RF) (Mohapatra et al., 2020), and kernel principal component analysis (KPCA) (Navi et al., 2018). These methods can effectively extract fault features to a certain extent, but there are some limitations. ML methods are unable to generate discriminative features of fault signal data, there methods always combined with the signal features extraction process, the features should be predesigned. However, predesigning handcrafted features is difficult. The features extraction process of fault signal is an exhausted work and impacts the fault diagnosis result. These methods require experts to have a rich mathematical knowledge about manual feature extraction, and the degree of automation of feature extraction is greatly limited. The method selected by the experts directly affects the diagnosis results.

As a branch of ML, deep learning (DL) has powerful functionality and flexibility. DL does not need to rely on expert experience and manual feature extraction (Zhang W. et al., 2018), so many scholars have gradually introduced DL methods, such as the deep belief network (Shao et al., 2018; Wang et al., 2020), sparse autoencoders, and convolution neural networks (CNNs) (Wen et al., 2018; Wu and Zhao, 2018; Gou et al., 2020; Sun et al., 2020) into fault diagnosis processes. These methods can improve the accuracy of fault diagnosis, but there are some limitations. These methods require certain preconditions: sufficient sample data are required, and the training and test samples need to have the same data distribution. When the distributions of sample data are different, the performances of the above methods would drop. They does not consider the use of fault data under different environments for fault diagnosis.

The concept of transfer Learning (TL) was first proposed in 1995 at a conference on neural information processing systems (Thrun, 1995). TL is adopted to improve the performance of the current task by using data, models, or tasks from the source task that are different from (but similar to) the target task (Pan and Yang, 2009; Chen et al., 2019). When the data attributes and feature spaces in the source domain and the target domain are similar but not identical, previous learning experience is used to solve the crossing domain problem (Pan and Yang, 2009; Wen et al., 2017a). There are many scenarios of TL, such as multi-task learning (Caruan, 1997) and domain adaptation (Saenko et al., 2010). Model-based TL can use the pre training knowledge acquired in the source domain to transform and summarize the deep model (Donahue et al., 2014). As a new ML method, many scholars have started to introduce the TL method into the process of fault diagnosis under variable conditions (Wen et al., 2017b; Wu et al., 2020). However, this method is rarely used in gas sensor fault diagnosis.

In this paper, a gas sensor fault diagnosis method based on TL with LeNet-5 in a complex environment is proposed. A large set of gas sensor fault signal data under normal environmental conditions is adopted to train the traditional LeNet-5 model until an excellent performance is observed. However, it is difficult to obtain an amount of fault signal data due to the limitation of experimental conditions under complex environment, so the fault signal data is limited. The traditional model and parameters of the LeNet-5 can transfer to a new target task with a small amount of fault data using the TL method. The TL with LeNet-5 method could make use of gas sensor fault data from different environments, resulting in a better performance in complex environments. The benefits of this novel method improve the accuracy of fault diagnosis in complex environments by utilizing gas sensor fault signal data from normal and complex environments when only a small quantity of target fault data exists.

The remainder of this article is organized as follows. The second section introduces the theoretical fundamentals. In the third section, a novel model based on TL with LeNet-5 for hydrogen sensor fault diagnosis is introduced. The fourth section verifies the performance of the proposed method. The fifth section contains the conclusions and future researches.

## Theoretical Fundamentals

### CNNs and LeNet-5

CNNs are widely used in image processing. They consist of a convolutional layer, pooling layer, and full connection (FC) layer. The convolutional layer can extract features via a convolutional operation on the previous layers of different convolutional kernels. More features can be extracted by using multiple convolutional kernels. The pooling layer can sample the features extracted from the convolutional layer. The sampling method can be divided into two parts: maximum sampling and mean sampling. In this paper, the maximum sampling method is adopted. Each node of the FC layer is connected with all nodes of the previous layer, which are used to integrate the features extracted from the front edge (Wu and Zhao, 2018). The mathematical model of the CNN follows Equation (1).

(1) $$\begin{array}{r} x_{j}^{l}\;=\;f\left(\sum_{i\in M_{\text{j}}}^{}x_{j}^{l-1}\times k_{ij}^{l}+b_{j}^{l}\right) \end{array}$$

where *M*_j_ denotes the input characteristic graph, *k* denotes the convolution kernel, *b* denotes the network bias, $x_{j}^{1}$ denotes the *l* layer output, and $x_{j}^{l-1}$ denotes the *l* layer input. The calculation method for subsampling layer neurons follows Equation (2):

(2) $$\begin{array}{r} x_{j}^{l}\;=\;f\left(\beta_{\text{j}}^{l}down\left(x_{i}^{l-1}\right)+b_{j}^{l}\right) \end{array}$$

where $down\left(x_{i}^{l-1}\right)$ denotes the subsampling function and β denotes the network multiplicative bias. The CNN's output layer model follows Equation (3):

(3) $$\begin{array}{r} O=\left(b_{o}+w_{o}f_{v}\right) \end{array}$$

where *f*_*v*_ denotes the eigenvector, *b*_*o*_, *w*_*o*_ denotes the deviation vector and the weight matrix.

There are many CNN models; for example, GoogLeNet (Szegedy et al., 2015), AlexNet (Krizhevsky et al., 2017), and LeNet-5 (LeCun, 2015). As a classic CNN, LeNet-5 is widely used for handwritten digital character recognition (Tivive and Bouzerdoum, 2005) and fault diagnosis (Wen et al., 2018; Sun et al., 2020). LeNet-5 is a CNN with a gradient-based learning structure, and its input layer is an image with a size of 32 × 32 pixels. The typical LeNet-5 structure consists of two alternating convolutional layers, two pooling layers, and the two-layer FC artificial neural network. Compared with Alenet, GoogLenet, VGG16, and other CNN algorithms, LeNet-5 method has simple structure and high accuracy (Wen et al., 2018; Lu et al., 2019), and has achieved good results in hydrogen sensor fault diagnosis (Sun et al., 2020). Therefore, this study adpots LeNet-5 as classifier.

### Transfer Learning

TL is committed to transferring information of knowledge obtained on sufficient labeled data of a source domain to a small amount of data of a target domain. From the data volume, the source domain data are easy to obtain, the data samples are sufficient, the target domain data are difficult to obtain, and the data samples are very limited. When the content of previous learning and the content of new problems are similar, and the potential data share some common characteristics, the migration effect is significant. For example, it is easier for a person to learn to ride a motorcycle after learning to ride a bicycle. The domain and task follow Equations (4) and (5)

(4) $$\begin{array}{r} D=\left\{X,P\left(X\right)\right\} \end{array}$$

(5) $$\begin{array}{r} T=\left\{Y,P\left(Y/X\right)\right\} \end{array}$$

where *D* denotes the domain and *T* denotes the task. *X*, *Y* are the feature space and category space, respectively, and *P*(*X*), *P*(*Y*/*X*) are the marginal probability distribution and the conditional probability density, respectively. TL based on parameters migration is adopted in this paper; that is, some parameters are shared between the target domain model and the source domain model. Its purpose is to mine the prior distributions or parameters shared between the source domain and target domain.

## Proposed Model for Fault Diagnosis of Hydrogen Sensors Based on Tl With Lenet-5

In this section, a novel model of TL with LeNet-5 is proposed for the fault diagnosis of hydrogen sensors. Firstly, a method for preprocessing the raw signal of hydrogen sensors is adopted. Secondly, TL with the LeNet-5 method is proposed for gas sensor fault diagnosis.

### Hydrogen Sensor Fault Signal Pre-treatment

In this paper, the data preprocessing method we adopted changes the raw fault signal into a two-dimensional gray image conversion (Sun et al., 2020). We normalized the fault data. This method could not only realize end-to-end data conversion, but also eliminate the influence of expert experiences as much as possible without any predefined parameters. Supposing that the sensor fault signal is *L*(*n*), it follows Equation (6)

(6) $$\begin{array}{r} L\left(n\right),n\;=\;1,2,\cdot \cdot \cdot \cdot \cdot ,N\times M \end{array}$$

and the resolution of the two-dimensional gray image is *N* × *M* pixels, where *N* is the width and *M* is the height. To ensure the symmetry of *L[i]*, the MOL as the matrix of *L[i]* follows Equation (7)

(7) $$\begin{array}{r} MOL=\left[\begin{array}{c} L\left(1\right)\;\cdots \;L\left(N\right)\\ \vdots \;\ddots \;\vdots \\ L\left(\left(M-1\right)N+1\right)\;\cdots \;L\left(NM\right) \end{array}\right] \end{array}$$

**Figure 1:**
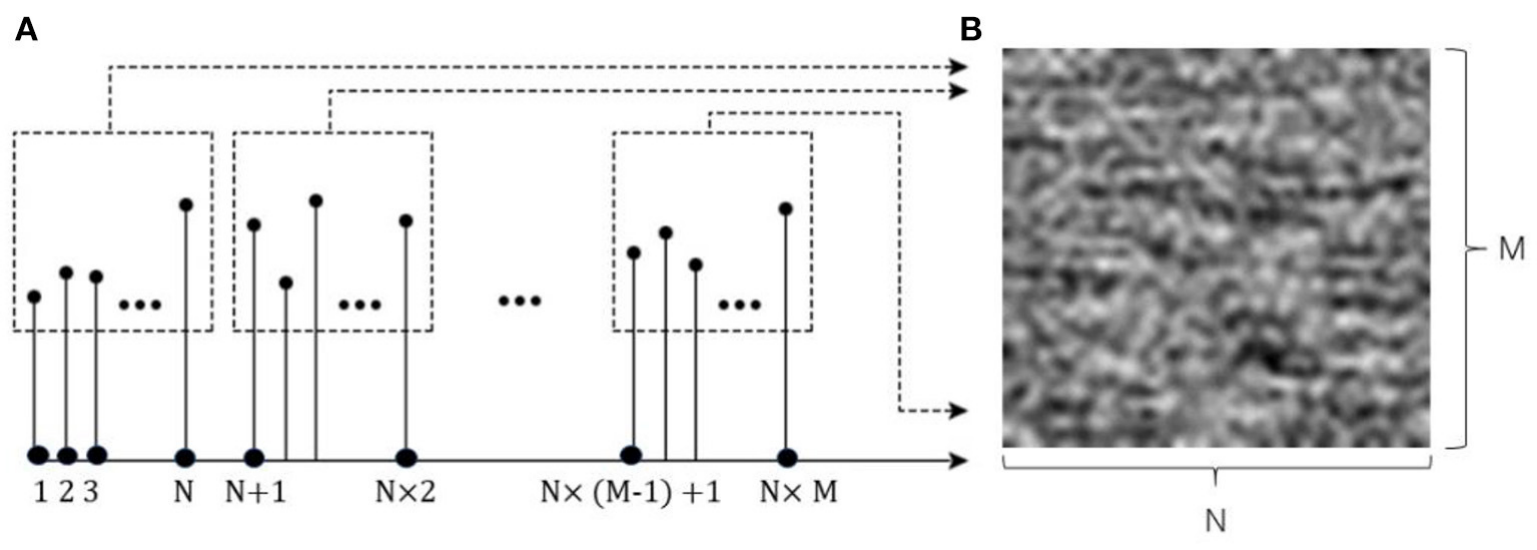
The process of gas sensor fault signal pretreatment. **(A)** One dimensional time domain gas sensor fault signal, **(B)** Two dimensional gray image.

The process of sensor fault signal pretreatment is shown in Figure 1.

### The Proposed Method of the TL With LeNet-5

**Figure 2:**
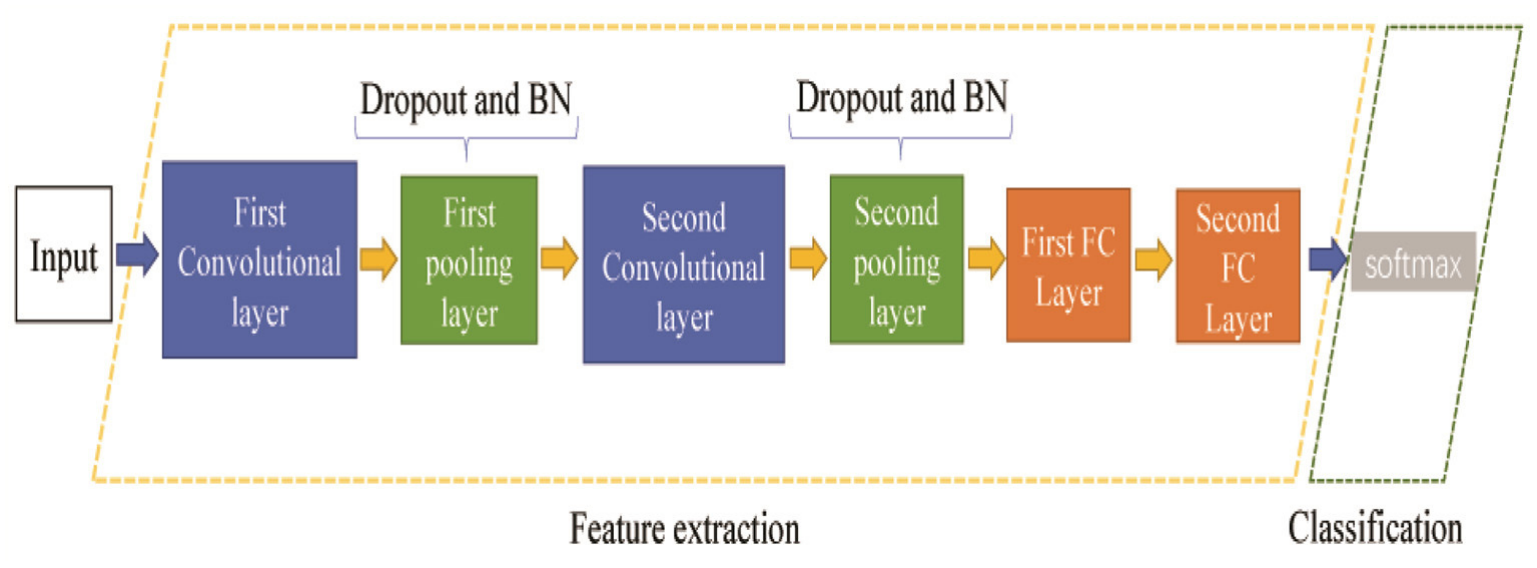
The LeNet-5 structure proposed in this paper.

Many CNN models have been proposed in recent years. This paper adopts the classic LeNet-5 model, which has been applied in many fields. The proposed LeNet-5 method consists of two parts: feature extraction and fault classification. It requires an image size of 32 × 32 pixels as the input; however, in order to improve the results of the gas sensor fault classification, we changed the size of the LeNet-5 input image. The revised width *N* of the gray image is 50 pixels, and the height *M* is 40 pixels. These adjustments depend on the volume of the raw fault signal and the architecture of feature extraction. The LeNet-5 consists of two convolution layers, two pooling layers, and two FC layers with two strategies: dropout and batch normalization (BN). The LeNet-5 structure proposed in this paper is shown in Figure 2.

This paper proposes TL with LeNet-5 method for gas sensor fault diagnosis in a complex environment, which involves two domains: the source domain and task domain. The source domain contains *S* kinds of gas sensor signal modes with sufficient fault data under a normal environment. The target domain contains *T* kinds of gas sensor signal modes with a small amount of fault data under a complex environment. The process of fault diagnosis is presented in six steps.

**Figure 3:**
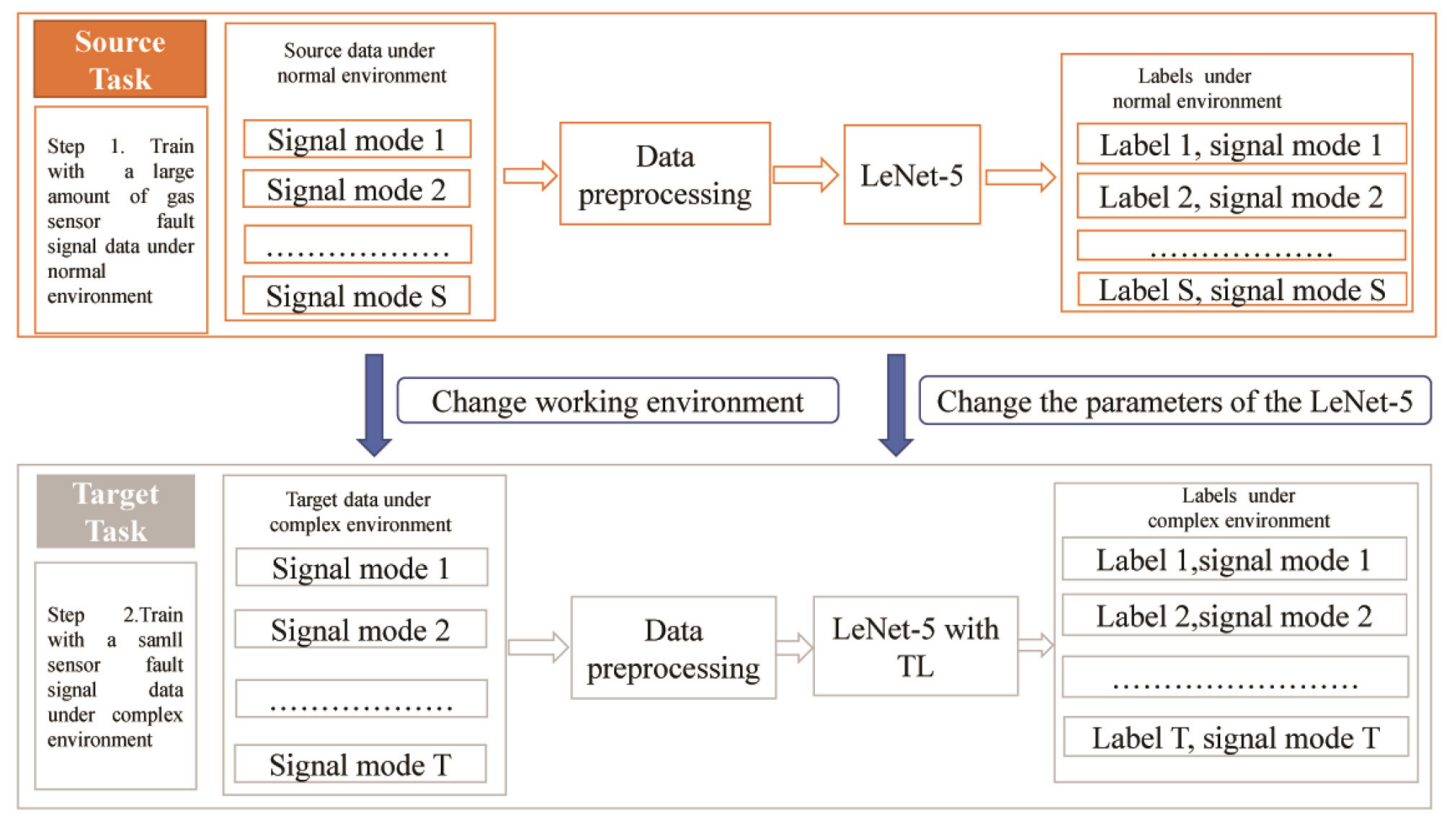
A detailed process of transfer learning with the LeNet-5-based gas sensor fault diagnosis model.

(1) *S* kinds of signal mode data in the source domain are preprocessed and converted into two-dimensional gray images.
(2) The images of source domain are input into the LeNet-5 model for training.
(3) The trained LeNet-5 model and parameters are transferred to the target domain.
(4) *T* kinds of signal mode data in the task domain are preprocessed and converted into two-dimensional gray images.
(5) The images of task domain are placed into the TL with LeNet-5 model for training, and the model parameters are fine tuned.
(6) The test sample data are adopted to test the trained model in order to verify the accuracy of the proposed method. The detailed process of the TL with LeNet-5-based gas sensor fault diagnosis model described in this paper is shown in Figure 3.

## Experiment and Validation of the Proposed Method

### Experimental Setup

Based on the environmental adaptability, reliability, and performance tests, together with the related literature, several typical fault signal modes of the SnO_2_ sensors are summarized in this study. These include heating wire disconnection (HWD), aging of the heating wire (AHW), aging of the sensitive body (ASB), exfoliation of the sensitive body (ESB), and false welding of the sensitive body (FWSB) (Sun et al., 2020). In order to obtain the data from five modes of fault signals under normal and complex environments, we collected fault data through a self-made special gas sensor test system. The test system is composed of a constant temperature and humidity box, a shaking table, an electric fan, a standard hydrogen concentration bottle, a standard air bottle, a gas molecular flow meter, a gas mixer, a bidirectional regulated power supply, a data collector, a computer, a temperature sensor, a humidity sensor, a sensor chamber, and six SnO_2_ sensor arrays.

**Figure 4:**
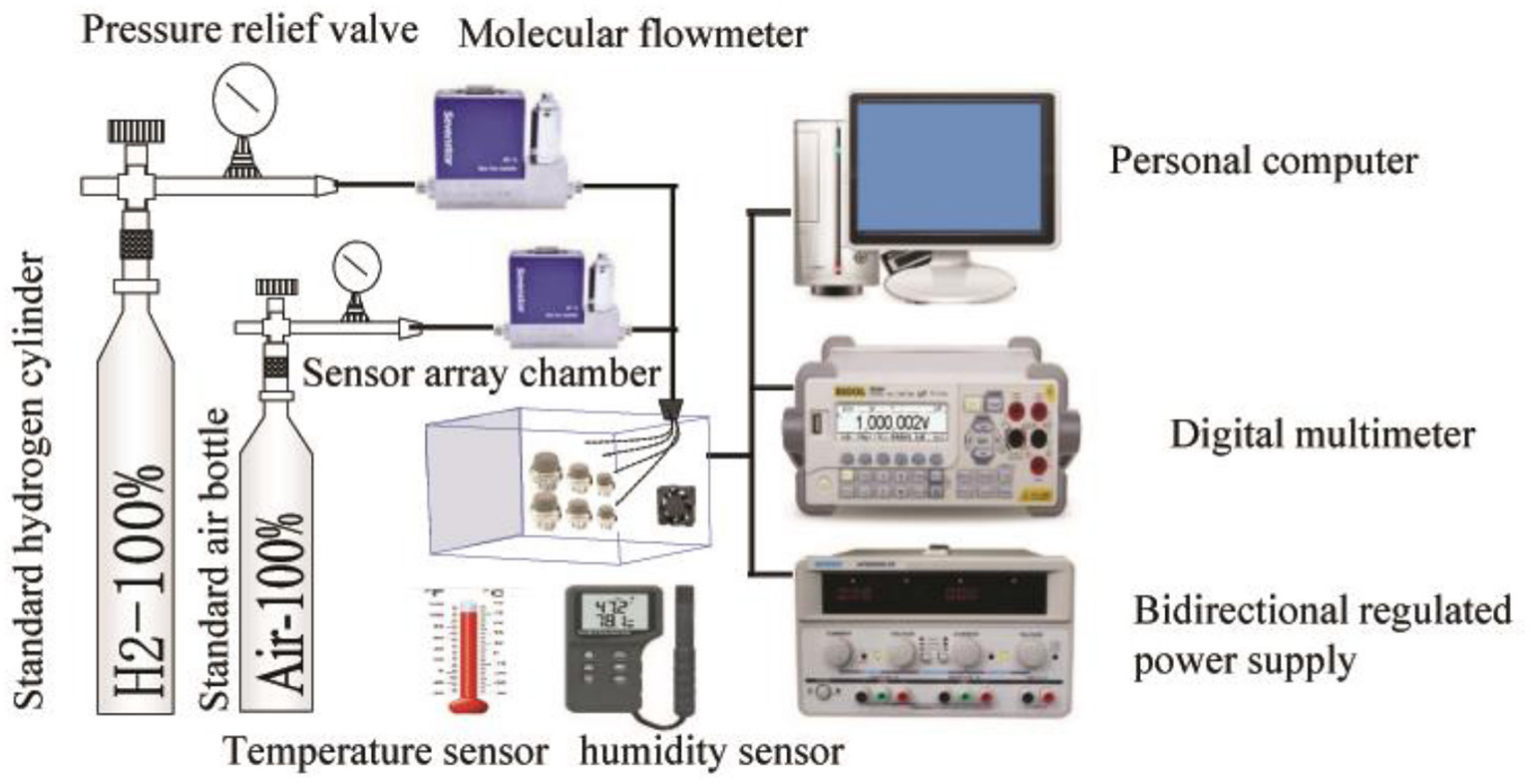
A system diagram of hydrogen sensor arrays.

The constant temperature and humidity box provided the test environment for temperature and humidity changes, the shaking table provided the test environment for vibration changes, and the electric fan provided the test environment for wind changes. The hydrogen sensor array system diagram is shown in Figure 4. A sensor array and gas chamber were placed into the constant temperature and humidity box and vibration table, respectively, to simulate temperature, humidity, and vibration variations in the environment. The fan was placed in the gas chamber to simulate wind changes in the test environment.

**Figure 5:**
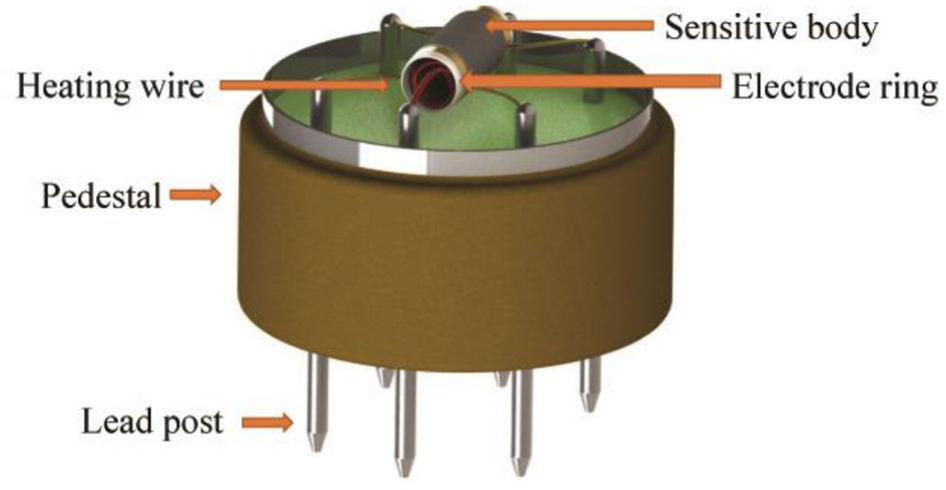
The MQ-8 sensor structure diagram.

**Figure 6:**
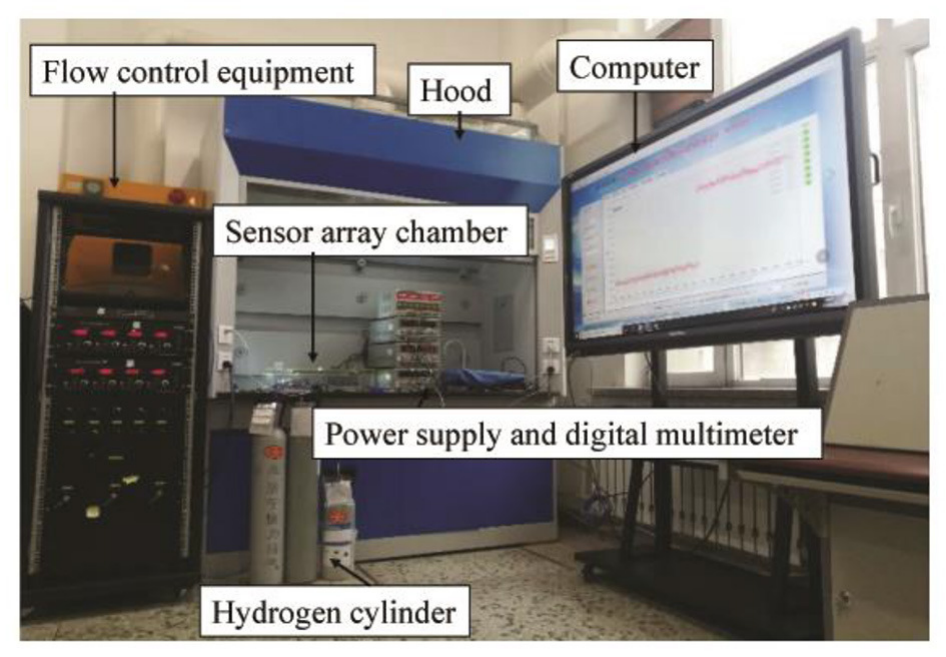
The experimental setup of MQ-8 gas sensor array.

The structure of the SnO_2_ sensor model (MQ-8) is shown in Figure 5. It is composed of a four-leg plastic base, nickel-plated copper column, stainless steel explosion-proof net, metal buckle ring, nickel-chromium heating wire, gas-sensitive body, gold lead, and porcelain tube carrier. The gold lead and the porcelain tube carrier were connected via gold slurry sintering welding, the nickel-chromium heating wire and the gold lead were connected via tin welding with the nickel-copper column, and the nickel-chromium heating wire and the gas-sensitive body were the key components of the hydrogen sensors. The nickel-chromium heating wire can provide a high-temperature working environment for the sensors. The function of the sensitive body was to detect the concentration of hydrogen and convert the value of the concentration into the resistance change. The function of the gold lead wire was to pass the information of the resistance change to the outside of the sensors through the nickel-copper column. The functional components of the gas sensor mentioned above are the main factors leading to the failure of the MQ-8 sensor. These variables keep constant during the experiment. The process of data acquisition is listed as follows: in the sensor array, each sensor has a separate power supply and a separate voltage divider. When the signals of the six sensors are collected, they are input to the single-chip microcomputer, integrated into the data acquisition card, and finally uploaded to the upper computer. The experimental device (the MQ-8 sensor array) is shown in Figure 6. The DL program was run on a 3.0 GHz Intel CPU and 8 GB RAM with Python 3.7.4 and TensorFlow 1.15.0 running Windows 10.

The general static calibration method of gas sensor is used to put the sensor into a standard gas box, and inject pure measured gas on the basis of the known space structure volume of the gas tank. After conversion, the standard gas concentration can be obtained. The standard gas concentration is placed on the sensor, and the sensor has output, then the standard gas concentration can be established the corresponding relationship with output can achieve the purpose of sensor calibration.

In this study, the normal environmental conditions are defined as a standard atmospheric pressure, temperature range between 17 to 23°C, humidity concentration range from 30 to 60%. The complex environmental conditions are defined as high humidity concentration range from 90 to 95%, low humidity concentration range from 10 to 20%, low temperature range from −10 to −30°C, high temperature range from 40 to 60°C. The wind is five meters per second, and it vibrates. The concentration range of hydrogen is 0.1–1%.

**Figure 7:**
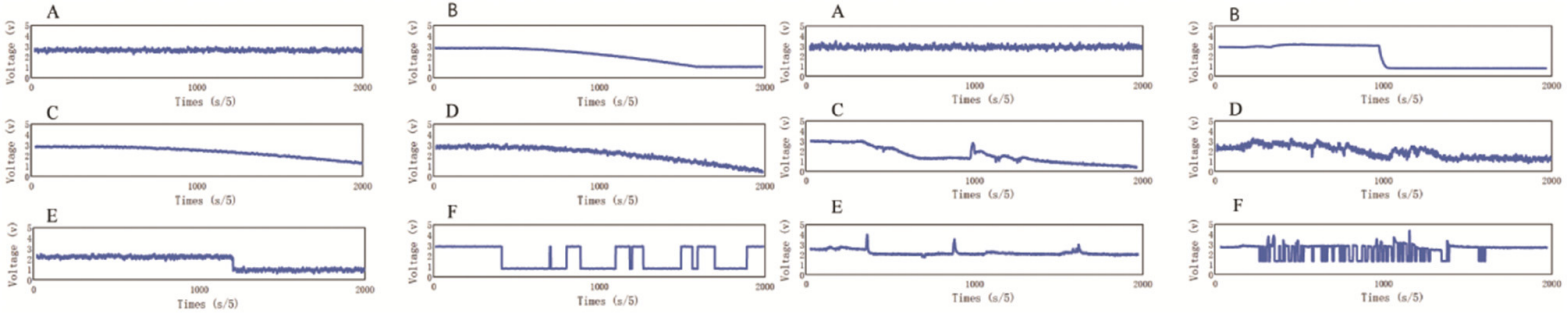
Six signal modes of MQ-8 gas sensor under a normal environment and complex environment. **(A)** Normal signal; **(B)** The signal including heating wire disconnection (HWD) fault; **(C)** The signal including aging of the heating wire (AHW) fault; **(D)** The signal including aging of the sensitive body (ASB) fault; **(E)** The signal including exfoliation of the sensitive body (ESB) fault; **(F)** The signal including false welding of the sensitive body (FWSB) fault.

The data from six signal modes (i.e., without fault and the five fault types) were obtained through the instrument and equipment we set up. We obtained the six signal modes of the MQ-8 sensor under a normal environment, as shown in Figure 7. The fault signal data were stable, so we used Matlab to simulate the six signal modes under a normal environment and increased the fault signal data number of the six signal modes. The sample data includes real samples and Matlab simulation samples under normal environment. The simulation data under different fault modes were obtained by the following ways: (1) The HWD fault was a linear signal with a larger slope which was superimposed on the normal output signal from a certain moment. The signal was stable at a certain value and at a certain moment. (2) The AHW fault was a linear signal with small slope superimposed at a certain moment of the normal output signal. (3) The aging cycle of ASB fault was long, so the aging process is accelerated in the simulation for the convenience of research, a linear signal with very small slope was superimposed on the normal signal from a certain time. (4) The ESB fault was to add a constant deviation data on the normal output signal from a certain time. (5) The output signal of the FWSB fault was 0 at a random time, and the output was normal at a certain time. On this basis, the white noise signal was superimposed.

Gas sensors often encounter complex environments in practice. In order to observe gas sensor fault signals in complex environments, we changed the temperature and humidity of a constant humidity incubator to increase the noise interference. The vibration noise interference could be increased by changing the vibration spectrum of the shaking table; the wind speed noise interference could be increased by the wind force generated by the electric fan in the gas chamber. In the end, the fault signals were obtained under complex environmental conditions. Therefore, the experiment under complex environment constructs a transformation environment, but carries on the measurement under the stable situation. Due to the complexity of the test conditions, we only get twenty-nine fault data for each fault type.

The six signal modes of the MQ-8 sensor under a complex environment are shown in Figure 7. As shown, the collected gas sensor fault signal changed greatly and was complex, so it was difficult to obtain these data through the Matlab simulation.

Through the experiments, the MQ-8 gas sensor signal mode types and samples of every type (i.e., the five fault types and without fault) under normal and complex environments were obtained to verify the effectiveness of TL with LeNet-5, as shown in Table 1.

**Table 1:** Signal mode types and samples of every type under normal and complex environments using transfer learning with LeNet-5.

	**Source task**	**Target task**
Working environments	Normal environment	Complex environment
Signal mode types	6	6
Samples of every type	100	29

### Validation of the TL With LeNet-5 Method

To validate the advantages of the proposed model in the fault diagnosis of a hydrogen sensor, tests were performed. The results of TL with LeNet-5 training and inference are presented in this section.

#### TL With LeNet-5 Training

There are data-rich sensor fault training samples under a normal environment in the source data compared with the target data under a complex environment. The LeNet-5 was trained and transferred from a normal environment to a complex environment. 100 samples of fault signal modes for each type in the source task were used to train the traditional LeNet-5. In the target task, only 20 samples of signal modes for each type were obtained to train the transferred LeNet-5. Nine samples of signal modes for each type in the target task were obtained for the test. The details of the labels and samples under normal and complex environmental conditions are shown in Table 2.

**Table 2:** Labels and samples under normal and complex environmental conditions.

**Label**	**Signals modes description**	**Normal environment conditions**	**Complex environmental conditions**	
		**Number of training samples**	**Number of training samples**	**Number of test samples**
1	Normal signal	100	20	9
2	HWD signal	100	20	9
3	AHW signal	100	20	9
4	ASB signal	100	20	9
5	ESB signal	100	20	9
6	FWSB signal	100	20	9

*The gas sensor signal data of six modes under normal environmental conditions were the source data of the transfer learning with LeNet-5 method. The data under complex environmental conditions were the target data of the transfer learning with LeNet-5 method. They were also the training data for other methods without transfer in the experiment*.

**Figure 8:**
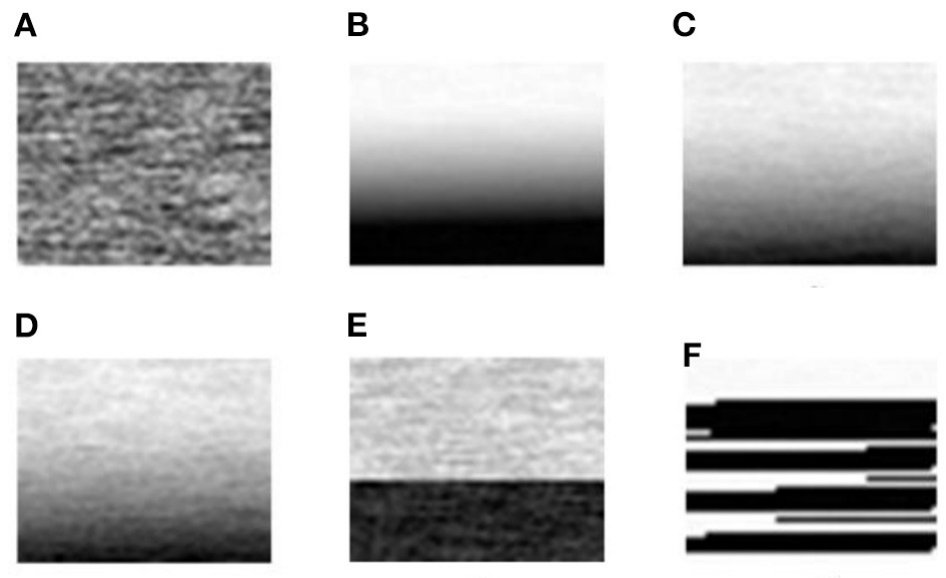
Converted two-dimensional gray images of the gas sensor signals of six modes under a normal environment. **(A)** Normal signal; **(B)** The signal including heating wire disconnection (HWD) fault; **(C)** The signal including aging of the heating wire (AHW) fault; **(D)** The signal including aging of the sensitive body (ASB) fault; **(E)** The signal including exfoliation of the sensitive body (ESB) fault; **(F)** The signal including false welding of the sensitive body (FWSB) fault.

**Figure 9:**
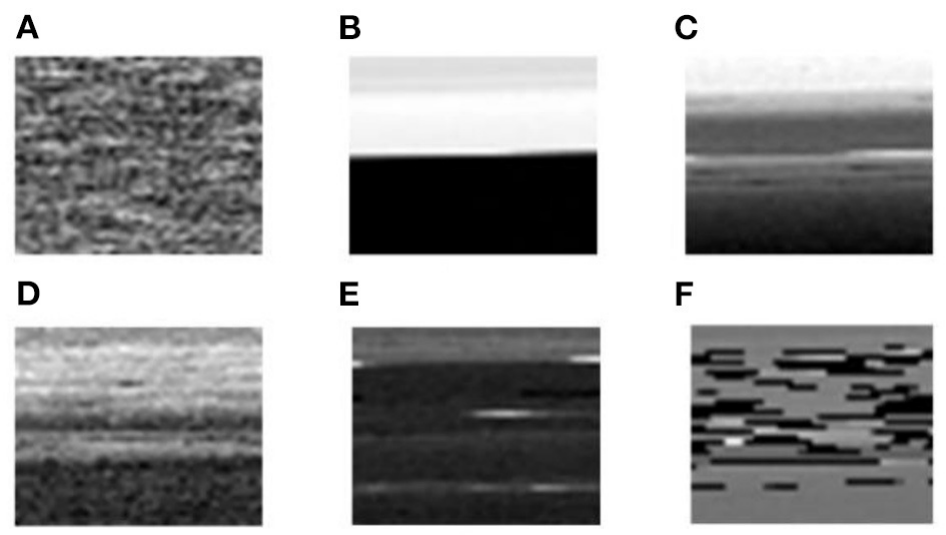
Converted two-dimensional gray images of the gas sensor signals of six modes under a complex environment. **(A)** Normal signal; **(B)** The signal including heating wire disconnection (HWD) fault; **(C)** The signal including aging of the heating wire (AHW) fault; **(D)** The signal including aging of the sensitive body (ASB) fault; **(E)** The signal including exfoliation of the sensitive body (ESB) fault; **(F)** The signal including false welding of the sensitive body (FWSB) fault.

As shown in Figures 8, 9, the six sensor signal modes under normal and complex environments were converted into two-dimensional gray images, and the size of each image was 50 × 40 pixels.

**Figure 10:**
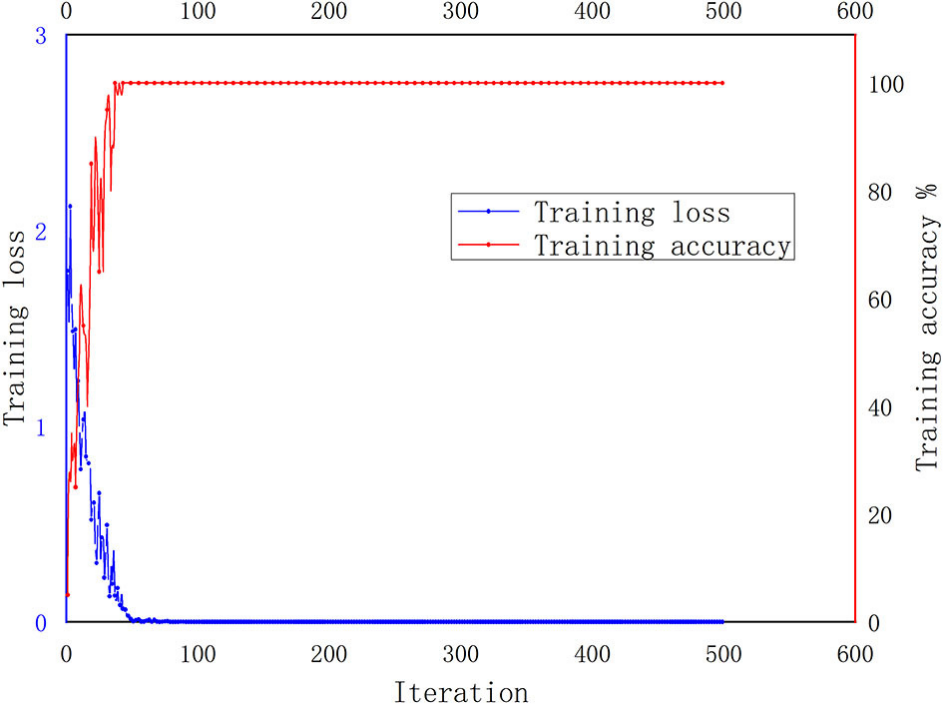
The LeNet-5 method's training accuracy and loss from the source task under a normal environment.

The two-dimensional gray images under a normal environment were input into the traditional LeNet-5 for training, and the number of experimental samples was 100 sets. The traditional LeNet-5 was trained for 500 iterations. As can be seen from Figure 10, the training accuracy reached 100%, and the training loss was close to 0 after about 50 iterations.

**Figure 11:**
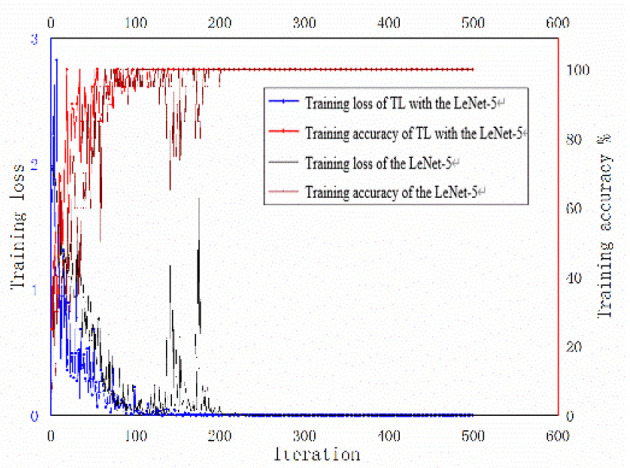
Training accuracy and loss of transfer learning with the LeNet-5 method and the traditional LeNet-5 method under a complex environment for the target task.

In order to verify the effectiveness of TL with LeNet-5 method, two methods were used to train the gray images. As shown in Figure 11, firstly, the traditional LeNet-5 model and parameters, which were trained in the source task, were transferred to the target task. The two-dimensional gray images under a complex environment were used as the target domain data for retraining. The TL with LeNet-5 method was trained for 500 iterations. The training accuracy reached 100%, and the training loss was close to 0 after about 150 iterations. Secondly, the two-dimensional gray images under a complex environment were input into the traditional LeNet-5 directly. The traditional LeNet-5 was trained for 500 iterations. The training accuracy reached 100%, and the training loss was close to 0 after about 200 iterations. The two methods both used 20 sets of experimental samples.

#### TL With LeNet-5 Inference

**Figure 12:**
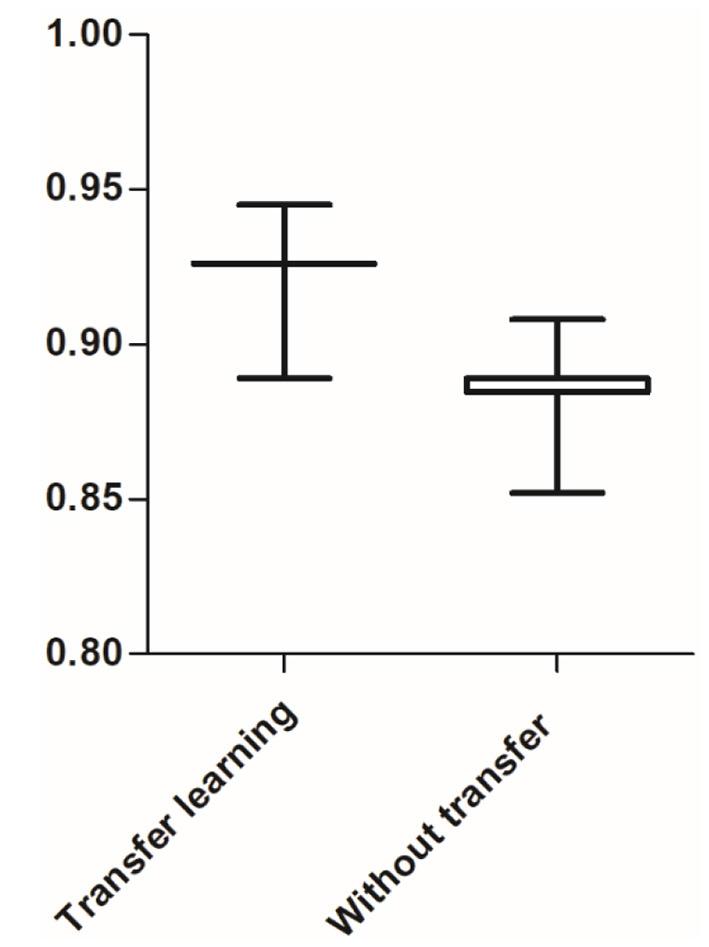
Boxplot of fault diagnosis accuracy of the different methods.

To obtain better results, the TL with LeNet-5 used cross-validation method. The experiments repeated 30 times. The diagnosis results of TL with LeNet-5 compared with the results of the traditional LeNet-5 without transfer, compared under a complex environment (in terms of accuracy). The total fault diagnosis accuracy of the traditional LeNet-5 was 88.48 ± 1.04%, while the total fault diagnosis accuracy of TL with LeNet-5 was 92.49 ± 1.28%. All the results of the fault diagnosis accuracy for different signal modes are shown in Table 3. The boxplot of total fault diagnosis accuracy is shown in Figure 12.

**Table 3:** Fault diagnosis accuracy of the different methods.

**Signals modes description**	**From normal environment to complex environment**		
	Without transfer (%)*	Transfer learning (%)*	Improvements (%)*
Normal signal	100.00 ± 0.00	100.00 ± 0.00	0.00 ± 0.00
HWD signal	88.53 ± 2.03	98.89 ± 3.39	10.36 ± 4.05
AHW signal	87.42 ± 4.82	88.90 ± 2.92	1.48 ± 3.84
ASB signal	88.16 ± 2.82	89.64 ± 2.82	1.48 ± 4.82
ESB signal	77.43 ± 2.03	78.54 ± 2.82	1.11 ± 3.39
FWSB signal	89.27 ± 2.03	99.26 ± 2.82	9.99 ± 3.39
Total	88.48 ± 1.04	92.49 ± 1.28	4.01 ± 1.61

$*\bar{X}\;\pm \;SD$.

### Discussion

In this study, the experimental conditions are limited, and only twenty-nine fault data are available for each fault under complex environment. The accuracy of fault diagnosis can be improved by using TL with LeNet-5 method. As shown in Figure 11, the convergence of the accuracy and the loss of the TL with LeNet-5 training were faster compared with the traditional LeNet-5 method without transfer. As can be seen in Table 3, transferring to different target task results in different performance. That is to say, the similarities of source task and target task could affect the performance of transfer learning.

Two other methods (Zhang P. et al., 2018) were added to comprehensively evaluate the performance: (1) using only the samples from normal environment to train the LeNet-5 model, and the same testing data as in the TL method were tested. (2) Using both the samples from the normal environment and the complex environment to directly train the LeNet-5 model (Without TL), and the same testing data as in the TL method were tested. The diagnostic results were 87.05% and 90.75%, respectively.

We also compared the TL with LeNet-5 method to traditional ML methods, such as LVQ (Bassiuny et al., 2007), ELM (the kernels is 116) (Song et al., 2019), SVM (the gamma value of polynomial kernel is 2) (Hu et al., 2005), KNN (Yang et al., 2016b), and RF (Mohapatra et al., 2020). All the experiments repeated 30 times, respectively, and all the results are presented in Table 4. The novel method had a higher accuracy than the traditional ML methods in a complex environment.

**Table 4:** Diagnosis accuracy based on seven different methods under a complex environment.

**Methods**	**Accuracy (%)**
LVQ	77.48 ± 1.12
ELM	79.50 ± 0.48
SVM	87.10 ± 0.92
KNN	85.19 ± 0.50
RF	88.01 ± 0.58
LeNet-5	88.48 ± 1.04
TL with LeNet-5	92.49 ± 1.28

## Conclusions and Future Researches

In this paper, a novel TL with LeNet-5 method was proposed for gas sensor fault diagnosis. The novel method has been validated by our self-made experimental system dataset. Traditional LeNet-5 without TL and other traditional ML methods were adopted for comparison.

In practice, there are usually abundant fault signal data under normal environmental conditions and limited fault signal data under complex environmental conditions. Furthermore, fault signal data in normal and complex environments might have different distributions. LeNet-5 improves the fault diagnosis accuracy of gas sensors in the same environment where the training data are abundant; however, it is not suitable for fault diagnosis in complex environments with limited training data. The experimental results show that the TL with LeNet-5 method could improve the accuracy of the fault diagnosis compared with the LeNet-5 without TL method and other traditional ML methods, which cannot take advantage of fault signal data in different distributions. The proposed method can provide a good fault diagnosis scheme for hydrogen sensors when only a small amount of fault data existing under complex environment.

The limitations of the proposed method is that, the common hydrogen sensor signal modes are needed to be represented in the dictionary list type. Otherwise the signal modes which have not been learned would be misclassified to be the known ones. Based on the limitation, the method can be modified to find an unknown signal mode in our future research work.

## Data Availability Statement

The original contributions presented in the study are included in the article/supplementary material, further inquiries can be directed to the corresponding author.

## Author Contributions

YS designed the research and wrote the first draft of the manuscript. HZ helped to organize the manuscript. TZ, ZZ, and BS processed the data. HZ, SL, YY, and SZ revised the final version. The work presented in this study was carried out by all authors in collaboration with one another. All authors have read and approved the final manuscript.

## Conflict of Interest

The authors declare that the research was conducted in the absence of any commercial or financial relationships that could be construed as a potential conflict of interest.

## References

[B1] Bassiuny A. Li X. Du R. (2007). Fault diagnosis of stamping process based on empirical mode decomposition and learning vector quantization. Int. J. Mach. Tools Manuf. 47, 2298–2306. 10.1016/j.ijmachtools.2007.06.006

[B2] Brown D. Lewis C. Weinberger B. I. (2015). Human exposure to unconventional natural gas development: a public health demonstration of periodic high exposure to chemical mixtures in ambient air. J. Environ. Sci. Health Part A 50, 460–472. 10.1080/10934529.2015.992663 25734822

[B3] Caruan R. (1997). Multitask learning. Mach. Learn. 28, 41–75. 10.1023/A:1007379606734

[B4] Chalk S. Miller J. F. (2006). Key challenges and recent progress in batteries, fuel cells, and hydrogen storage for clean energy systems. J. Power Sources 159, 73–80. 10.1016/j.jpowsour.2006.04.058

[B5] Chen X. Wang S. Fu B. Long M. Wang J. (2019). “Catastrophic forgetting meets negative transfer: batch spectral shrinkage for safe transfer learning,” in Advances in Neural Information Processing Systems 32, eds H. Wallach, H. Larochelle, A. Beygelzimer, F. d'Alché-Buc, E. Fox, and R. Garnett (Red Hook, NY: Curran Associates, Inc.), 1906–1916.

[B6] Chen Y. Yang J. Xu Y. Jiang S. Liu X. (2016). Status self-validation of sensor arrays using gray forecasting model and bootstrap method. IEEE Trans. Instrum. Meas. 65, 1626–1640. 10.1109/TIM.2016.2540942

[B7] Chen Y. S. Xu Y. H. Yang J. L. Shi Z. Jiang S. D. Wang Q. (2016). Fault detection, isolation, and diagnosis of status self-validating gas sensor arrays. Rev. Sci. Instrum. 87:045001. 10.1063/1.4944976 27131696

[B8] Donahue J. Jia Y. Vinyals O. Hoffman J. Zhang N. (2014). “A deep convolutional activation feature for generic visual recognition,” in International Conference on Machine Learning (ICML) (Beijing), 647–655.

[B9] Fedorenko G. Oleksenko L. Maksymovych N. Skolyar G. Ripko O. (2017). Semiconductor gas sensors based on pd/so2 nanomaterials for methane detection in air. Nanoscale Res. Lett. 12:329. 10.1186/s11671-017-2102-0 28476083 PMC5418166

[B10] Gou L. Li H. Zheng H. Li H. Pei X. (2020). Aeroengine control system sensor fault diagnosis based on cwt and cnn. Math. Probl. Eng. 2020:5357146. 10.1155/2020/5357146

[B11] Hu Z. H. Cai Y. Z. Li Y. G. Xu X. M. (2005). Data fusion for fault diagnosis using multi-class support vector machines. J. Zheijang Univ. Sci. A 6, 1030–1039. 10.1631/jzus.2005.A1030

[B12] Ingimundarson A. Stefanopoulou A. G. McKay D. A. (2008). Model-based detection of hydrogen leaks in a fuel cell stack. IEEE Trans. Control Syst. Technol. 16, 1004–1012. 10.1109/TCST.2007.916311

[B13] Krizhevsky A. Sutskever I. Hinton G. E. (2017). Imagenet classification with deep convolutional neural networks. Commun. ACM 60, 84–90. 10.1145/3065386

[B14] LeCun Y. (2015). LeNet-5, Convolutional Neural Networks. Available online at: https://yann.lecun.com/exdb/lenet.

[B15] Lu S. Qian G. He Q. Liu F. Liu Y. B. Wang Q. J. (2019). *In situ* motor fault diagnosis using enhanced convolutional neural network in an embedded system. IEEE Sens. J. 20, 8287–8296. 10.1109/JSEN.2019.2911299

[B16] Ma G. Li C. Luo Y. Mu R. Wang L. (2012). High sensitive and reliable fiber Bragg grating hydrogen sensor for fault detection of power transformer. Sens. Actuators B Chem. 169, 195–198. 10.1016/j.snb.2012.04.066

[B17] Mohapatra D. Subudhi B. Daniel R. (2020). Real-time sensor fault detection in tokamak using different machine learning algorithms. Fusion Eng. Des. 151:111401. 10.1016/j.fusengdes.2019.111401

[B18] Navi M. Meskin N. Davoodi M. (2018). Sensor fault detection and isolation of an industrial gas turbine using partial adaptive kpca. J. Process Control 64, 37–48. 10.1016/j.jprocont.2018.02.002

[B19] Pan J. Yang Q. (2009). A survey on transfer learning. IEEE Trans. Knowl. Data Eng. 22, 1345–1359. 10.1109/TKDE.2009.191

[B20] Poirier M. Sapundzhiev C. (1997). Catalytic decomposition of natural gas to hydrogen for fuel cell applications. Int. J. Hydrogen Energy 22, 429–433. 10.1016/S0360-3199(96)00101-2

[B21] Saenko K. Kuli B. Fritz M. Darrell T. (2010). “Adapting visual category models to new domains,” in European Conference on Computer Vision (ECCV) (Heraklion), 213–226.

[B22] Shao H. Jiang H. Zhang H. Liang T. C. (2018). Electric locomotive bearing fault diagnosis using a novel convolutional deep belief network. IEEE Trans. Ind. Electron. 65, 2727–2736. 10.1109/TIE.2017.2745473

[B23] Song K. Xu P. Chen Y. Zhang T. Wei G. Wang Q. (2019). A fault diagnosis and reconfiguration strategy for self-validating hydrogen sensor array based on mwpca and elm. IEEE Access 7,115075–115092. 10.1109/ACCESS.2019.2936128

[B24] Staffell I. Scamman D. Abad A. V. Balcombe P. (2019). The role of hydrogen and fuel cells in the global energy system. Energy Environ. Sci. 12, 463–491. 10.1039/C8EE01157E

[B25] Sun Y. Zhang H. Zhao T. Zou Z. Shen B. Yang L. (2020). A new convolutional neural network with random forest method for hydrogen sensor fault diagnosis. IEEE Access 8, 85421–85430. 10.1109/ACCESS.2020.2992231

[B26] Szegedy C. Liu W. Jia Y. Sermanet P. Reed S. Anguelov D.. (2015). “Going deeper with convolutions,” in Proceedings of the IEEE Conference on Computer Vision and Pattern Recognition (Boston, MA), 1–9.

[B27] Thrun S. (1995). “Extracting rules from artificial neural networks with distributed representations,” in Advances in Neural Information Processing Systems (Denver, CO), 505–512.

[B28] Tivive F. H. C. Bouzerdoum A. (2005). “An eye feature detector based on convolutional neural network,” in https://ieeexplore.ieee.org/xpl/conhome/10550/proceeding *Proceedings* of the Eighth International Symposium on Signal Processing and Its Applications (Sydney, NSW).

[B29] Tsujita W. Yoshino A. Ishida H. Moriizumi T. (2005). Gas sensor network for air-pollution monitoring. Sens. Actuators B Chem. 110, 304–311. 10.1016/j.snb.2005.02.008

[B30] Wang Y. Pan Z. Yuan X. Yang C. Gui W. (2020). A novel deep learning- based fault diagnosis approach for chemical process with extended deep belief network. ISA Trans. 96, 457–467. 10.1016/j.isatra.2019.07.001 31324340

[B31] Wen L. Gao L. Li X. (2017a). A new deep transfer learning based on sparse auto-encoder for fault diagnosis. IEEE Trans. Syst. Man Cybern. Syst. 49, 136–144. 10.1109/TSMC.2017.2754287

[B32] Wen L. Li X. Gao L. (2017b). A transfer convolutional neural network for fault diagnosis based on resnet-50. Neural Comput. Appl. 32, 6111–6124. 10.1007/s00521-019-04097-w

[B33] Wen L. Li X. Gao L. Zhang Y. (2018). A new convolutional neural network-based data-driven fault diagnosis method. IEEE Trans. Ind. Electron. 65, 5990–5998. 10.1109/TIE.2017.2774777

[B34] Winter C. J. (2005). Into the hydrogen energy economy-milestones. Int. J. Hydrogen Energy 30, 681–685. 10.1016/j.ijhydene.2004.12.011

[B35] Wu H. Zhao J. (2018). Deep convolutional neural network model based chemical process fault diagnosis. Comput. Chem. Eng. 115, 185–197. 10.1016/j.compchemeng.2018.04.009

[B36] Wu Z. Jiang H. Zhao K. Li X. (2020). An adaptive deep transfer learning method for bearing fault diagnosis. Measurement 151:107227. 10.1016/j.measurement.2019.10722733114173

[B37] Yang J. Chen Y. Zhang L. Sun Z. (2016a). Fault detection, isolation, and diagnosis of self-validating multifunctional sensors. Rev. Sci. Instrum. 87:065004. 10.1063/1.4954184 27370486

[B38] Yang J. Sun Z. Chen Y. (2016b). Fault detection using the clustering-kNN rule for gas sensor arrays. Sensors 16:2069. 10.3390/s16122069 27929412 PMC5191050

[B39] Zhang P. Ma X. Chen L. Zhou J. Wang C. (2018). Decoder calibration with ultra small current sample set for intracortical brainmachine interface. J. Neural Eng. 15:026019. 10.1088/1741-2552/aaa8a4 29343650

[B40] Zhang Q. Zhou Q. Lu Z. Wei Z. Xu L. Gui Y. (2018). Recent advances of SnO_2_-based sensors for detecting fault characteristic gases extracted from power transformer oil. Front. Chem. 6:364. 10.3389/fchem.2018.00364 30211152 PMC6123357

[B41] Zhang W. Li C. Peng G. Chen Y. Zhang Z. (2018). A deep convolutional neural network with new training methods for bearing fault diagnosis under noisy environment and different working load. Mech. Syst. Signal Process. 100, 439–453. 10.1016/j.ymssp.2017.06.022

